# In Vitro, In Vivo, and In Silico Models of Lymphangiogenesis in Solid Malignancies

**DOI:** 10.3390/cancers14061525

**Published:** 2022-03-16

**Authors:** Sophie Bekisz, Louis Baudin, Florence Buntinx, Agnès Noël, Liesbet Geris

**Affiliations:** 1 Biomechanics Research Unit, GIGA In silico Medicine, ULiège, 4000 Liège, Belgium; liesbet.geris@uliege.be; 2 Laboratory of Biology of Tumor and Development, GIGA Cancer, ULiège, 4000 Liège, Belgium; louis.baudin@uliege.be (L.B.); florence.buntinx@uliege.be (F.B.); agnes.noel@uliege.be (A.N.); 3 Biomechanics Section, KU Leuven, 3000 Leuven, Belgium; 4 Skeletal Biology and Engineering Research Center, KU Leuven, 3000 Leuven, Belgium

**Keywords:** lymphatic endothelial cells, cancer, metastatic dissemination, in vitro models, in vivo models, lymphangiogenesis, in silico methods, computational models

## Abstract

**Simple Summary:**

Lymphangiogenesis is the formation of new lymphatic vessels in physiological conditions but has also been found to be associated with pathologies. For example, it has been proven to be involved in cancer progression and metastatic dissemination through the body. Thus, it became a key element to study in the management of this widespread disease. To date, the study of lymphangiogenesis takes place at the biological (in vitro and in vivo) and computational (in silico) levels. The association of these complementary fields combined with imaging techniques constitutes a real toolbox in pathological lymphangiogenesis understanding.

**Abstract:**

Lymphangiogenesis (LA) is the formation of new lymphatic vessels by lymphatic endothelial cells (LECs) sprouting from pre-existing lymphatic vessels. It is increasingly recognized as being involved in many diseases, such as in cancer and secondary lymphedema, which most often results from cancer treatments. For some cancers, excessive LA is associated with cancer progression and metastatic dissemination to the lymph nodes (LNs) through lymphatic vessels. The study of LA through in vitro, in vivo, and, more recently, in silico models is of paramount importance in providing novel insights and identifying the key molecular actors in the biological dysregulation of this process under pathological conditions. In this review, the different biological (in vitro and in vivo) models of LA, especially in a cancer context, are explained and discussed, highlighting their principal modeled features as well as their advantages and drawbacks. Imaging techniques of the lymphatics, complementary or even essential to in vivo models, are also clarified and allow the establishment of the link with computational approaches. In silico models are introduced, theoretically described, and illustrated with examples specific to the lymphatic system and the LA. Together, these models constitute a toolbox allowing the LA research to be brought to the next level.

## 1. Introduction

Cancer is still one of the major causes of death worldwide, and the incidence of this group of diseases will continue to grow because of the increasing ageing of the population [1]. A better understanding of this malignancy and its different features is required and essential to develop treatments or at least to allow this disease to become more chronic and non-lethal. Normal and healthy cells turning malignant share common identified traits and characteristics, well known as the hallmarks of cancer [2]. Tumor-induced vasculature belongs to these proven features. Excessive angiogenesis and lymphangiogenesis (LA) are associated with cancer progression and bad prognosis [3,4,5]. Indeed, both angiogenesis and LA first enable tumor cells to have access to nutrients, oxygen, and waste disposal and then allow them to disseminate to distant parts of the body and form metastases through the circulatory system [6,7,8,9]. Although tumor angiogenesis has already been investigated intensively and anti-angiogenic drugs developed, interest in tumor LA is fairly recent, even though its importance has been proven many times [10,11].

LA (vs angiogenesis) is the formation of new lymphatic (vs. blood) vessels from pre-existing ones. Although quiescent in normal conditions due to its development being restricted almost exclusively to the embryonic or postnatal stages, lymphatic vasculature can undergo intense remodeling under pathological conditions [12,13,14,15]. This remodeling consists in abnormal lymphatic dysplasia, vessel dilatation, and increased permeability, which can be or not be associated with LA. Pathological LA is reported in inflammation [16,17], wound healing [18,19], graft transplant rejection [20,21], fibrosis [22], lymphedema, which often results from cancer treatments [23,24], cancer [25], etc. [26,27]. While LA can be beneficial during the first stage of cancer progression in draining inflamed tissues and allowing immune cells tumor antigens to circulate, it is also responsible for the metastatic spread to the lymph nodes (LNs) and thereafter the dissemination to distant organs [6,28]. Indeed, depending on the cancer type, the primary tumor secretes pro-angiogenic and/or pro-lymphangiogenic growth factors to promote angiogenesis and/or LA. Cancer cells can therefore use blood and/or lymphatic route(s) to disseminate and form metastases [29,30]. In breast, cervical, and prostate cancers and melanoma, excessive LA is associated with the cancer progression and metastatic dissemination to the LNs through the lymphatic vessels [31]. The primary tumor actually also conditions the draining LNs to host cancer cells through a pre-metastatic remodeling and the establishment of a niche even before the cancer cells reach them [32]. LN metastases have thereafter the potential to seed distant organs [8,9,33,34]. Confirming the importance of the lymphatic route, the targeting of the VEGF-C or VEGFR-3 for therapeutic purposes demonstrates its worth for the anti-lymphangiogenic and combined treatments under development [35,36,37]. The surgical dissection of LNs when cancerous or for prevention and diagnosis can lead to the formation of lymphedemas, characterized by localized swellings [38,39,40]. The actual existing treatments for lymphedemas (surgery, compression, and draining massages) are unfortunately not very effective [41]. A better understanding of LA through cancer research could be translated to the abnormalities of lymphatic vessels in lymphedemas and therefore be of great interest in improving the lifestyles of patients with such disability.

The experimental models and biological markers of LA are of paramount importance in studying this process and how it is involved in tumorigenesis [42,43]. Since the discovery of valuable markers [44], increasingly accurate in vitro and in vivo models have been developed to represent LA, or at least parts of it, in a healthy or pathological context [45]. Both the in vitro and the in vivo models have their own particular characteristics, advantages, and drawbacks. By definition, a model is a simplification of the reality and cannot display all the specificities of the process under study. Highlighting only some angles, models need to be combined to investigate the whole process. Although more relevant and representative than in vitro ones, in vivo models are often more complicated to set up due to financial, timing, and ethical constraints. In vitro models are nonetheless continuously improved towards three-dimensional (3D) and multicellular configurations to better match the reality and to complement the in vivo ones. More recently, mathematical and computational models, referred as in silico, have been implemented in the context of cancer, including tumor vasculogenesis, and their contributions are not negligible when put together with the in vitro and in vivo models [46,47]. In addition to their remarkable integration capacity, numerical models are also able to perform large-scale screening experiments, guide scientists towards the most supposedly impactful experiments, and generate new out-of-the-box insights [48,49].

This paper aims at reviewing the published literature on the in vitro, in vivo, and in silico models of LA, mainly in the context of cancer progression and dissemination. First, a short reminder about this process in healthy and pathological conditions will be presented. Then, the different models, their principal features, and their pros and cons will be explained, with a clear focus on their scopes of application and the represented space–time scale. The in vitro and in vivo models are discussed first, including an overview of the in vivo imaging techniques. Subsequently, the in silico models will be reviewed. Given the unfortunately still limited use of in silico models in LA research, some technical reminders about mathematical and computational modeling are explained. Although in vitro and in vivo models precisely related to tumor LA are quite current, the correlated in silico models are less common and are therefore supplemented, in this paper, with mathematical and computational models of the lymphatic system in general. Finally, the limitations and perspectives in the field of LA modeling will be discussed.

## 2. Biological Reminders of Molecular and Cellular Lymphangiogenesis

**Figure 1:**
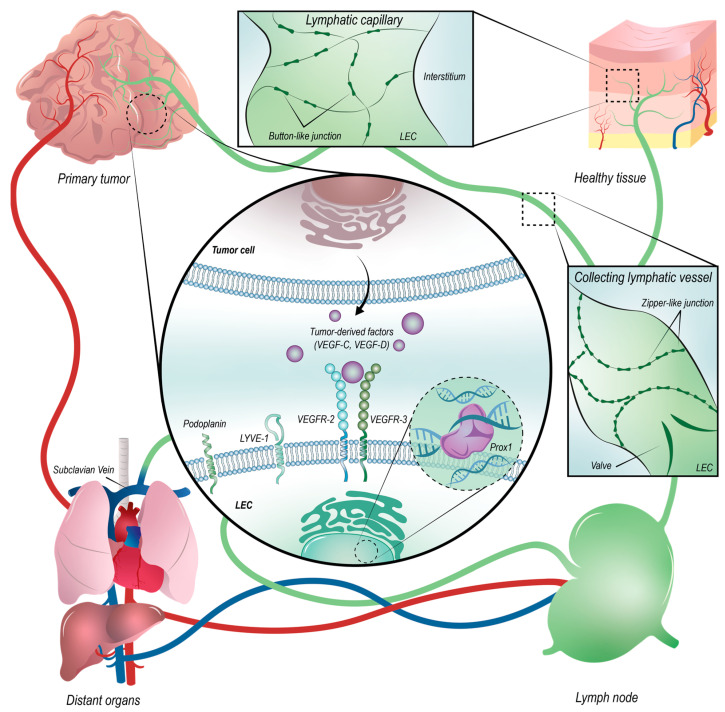
Lymphatic system organization and lymphatic marker location. The lymph first collected by lymphatic capillaries displaying button-like junctions transits in pre- and collecting lymphatic vessels characterized by zipper-like junctions and valves, then in lymph nodes (LNs), and finally returns to the bloodstream via the subclavian veins. In some cancers, the primary tumor promotes lymphangiogenesis by secreting pro-lymphangiogenic vascular endothelial growth factors such as VEGF-C and-D, which interact with the specific lymphatic vascular endothelial growth factor receptors VEGFR-2 and -3. Cancer cells can use either the blood circulation or the lymphatic route to disseminate to distant organs through the LNs. The membrane VEGFR-3, LYVE-1 (lymphatic vessel endothelial hyaluronan receptor), and podoplanin, as well as the nuclear Prox1 (prospero homeobox protein 1), are the main markers of LECs.

The lymphatic system is essential for maintaining tissue fluid homeostasis, as well as for absorbing and transporting fatty acids. It is also involved in immune response by transporting immune cells and soluble antigens from peripheral tissues towards the lymph nodes [50,51]. Lymphatic vessels are found in all vascularized tissues, except in bone marrow and cornea. Contrary to the blood circulation, it is an open system. The overflow of fluid is first reuptaken by initial lymphatic vessels, which are small blind-ended capillaries with an incomplete basement membrane deprived of pericytes and smooth muscle cells. In capillaries, lymphatic endothelial cells (LECs) are joined through discontinuous “button-like” cell-cell junctions, enabling fluid and other cells to enter into the lymphatic vessel lumen (Figure 1). Fluids are then transported through pre- and collecting lymphatics, which are characterized by a complete basement membrane, smooth muscle cells, valves, and zipper-like junctions preventing lymph leak. After being passed through the LNs, the collecting vessels reach the thoracic duct and the lymph is returned in the bloodstream through terminal lymphatics via the subclavian veins [25,52].

Lymphangiogenesis is the counterpart of angiogenesis for the lymphatic vessels and is therefore the formation of new lymphatic vessels by LECs sprouting from pre-existing lymphatic vessels. This process involves LEC proliferation, migration, and survival, which are essentially driven and stimulated by lymphangiogenic actors, among which the major ones are the vascular endothelial growth factor receptor-2 and -3 (VEGFR-2, VEGFR-3), and their ligands: vascular endothelial growth factor-C and -D (VEGF-C, VEGF-D) [53,54]. In response to these growth factors, these cells acquire an invasive phenotype and migrate by developing long filopodia. Different cells are involved in the process of LA: cells with an invasive character, which will migrate and degrade the extracellular matrix (ECM), and cells with a proliferative character, which allow the growth of lymphatic vessels. Even if BECs and LECs present morphological similarities and a similar formation of new vessels, there are however differences which do not allow a direct transposition [55]. In particular, the endocytic receptor uPARAP is present in LECs but totally absent in BECs [56].

Many advances have been made possible on the lymphatic system and therefore on the LA by the discovery of specific markers for LECs, which are different from the blood cell ones. Among these markers, it is possible to distinguish the VEGFR-3 involved in various LEC signaling pathways [53,57], the lymphatic vessel endothelial hyaluronan receptor (LYVE-1) [58], the mucin-like glycoprotein podoplanin (PDPN) [59], and the prospero homeobox protein 1 (Prox1) [60] (Figure 1). Prox1 is a predominant lymphatic transcription factor and therefore preferentially located in the nucleus which contributes to the LEC’s fate. Despite their expression in LECs, a remaining lack of specificity exists and the perfect marker for lymphatic vessels has unfortunately not been identified yet. Indeed, given that they are also expressed in other cellular types [28,50], these markers are usually combined to specifically characterize LECs in situ and to isolate them for in vitro studies.

Even if quiescent in normal conditions, LA takes place in several pathologies [16,17,18,19,20,21,22,23,24,26,27], including cancer [25]. While LA can be beneficial during the first stage of cancer progression, it is also responsible for metastatic spread to the LNs and thereafter the dissemination to distant organs [6,28]. In certain types of cancer, excessive LA is associated with cancer progression and metastatic dissemination to the LNs through the lymphatic vessels [31]. The primary tumor actually secretes pro-lymphangiogenic growth factors and conditions the draining LNs to host cancer cells through a pre-metastatic remodeling and the establishment of a niche even before the cancer cells reach them [32]. Whether the cancer cells in LNs can then seed distant metastases to other organs has been a subject of considerable debate. Certain experts viewed LN metastases as clinically inconsequential [61,62], whereas other experts considered them to have the potential to seed distant organs [8,9,33,34]. These final data/references provide a definitive proof-of-concept that metastatic cells in LNs can seed distant organs and are not only worth considering but important to treat (at least for specific cancer (sub)types).

Anti-angiogenic drugs are used successfully in the clinic to treat some advanced cancers, but they are associated with side effects and the development of resistance [63]. Given that these currently used anti-angiogenic drugs do not interfere with LA, one possibility for improving cancer treatments focusing on vasculature would be to target tumor LA, in combination or not with tumor angiogenesis. VEGF-C/VEGFR-3 are two important therapeutic targets in the anti-lymphangiogenic therapies under development [35,36]. Moreover, encouraging data have been reported for the combination of anti-angiogenic and anti-lymphangiogenic treatments [37]. Unfortunately, very few drugs specifically targeting LA have entered clinical testing so far [35,36], and none of them are FDA approved yet.

## 3. In Vitro Models

The culture of LEC results from the isolation of blood endothelial cells (BECs) and LECs from tissue. These endothelial cells (BECs and LECs) can be harvested from macrovessels such as the aorta or the lymphatic duct [64]. However, the use of this type of cells is not very meaningful in the case of lymphangiogenic studies as the new vessels come from the microvascularization and not from the macrovascularization. Another method of isolation consists in collecting capillary endothelial cells from the tissues where they are most frequently found. LECs are mainly derived from the skin, which incidentally represents the most important source of LECs used (primary LECs). Other LECs are derived from rats (RMLECs) [65] or from patient lymphangiomas. In order to discriminate BECs from LECs, immunopurification is performed using specific markers, and the cells are sorted by Fluorescent Activated Cell Sorter (FACS) or via magnetic beads. In this case, the markers related to LECs are usually CD31/podoplanin or CD31/LYVE-1. However, this discrimination method does not prevent the contamination of LECs by BECs in view of the expression profiles. Indeed, BECs can express lymphatic markers or can differentiate into LECs. To overcome this selection issue, it is possible to obtain LECs by differentiating progenitor cells (embryonic stem cells) into this specific lineage. Note that the dedifferentiation remains a notorious problem. Despite the fact that most of the used cells are primary LECs, different strategies exist to transform and immortalize them. The first strategy consists in using cells from transgenic animals. The second strategy allows the transformation of LECs into an LEC line containing human telomerase reverse transcriptase (hTERT).

**Figure 2:**
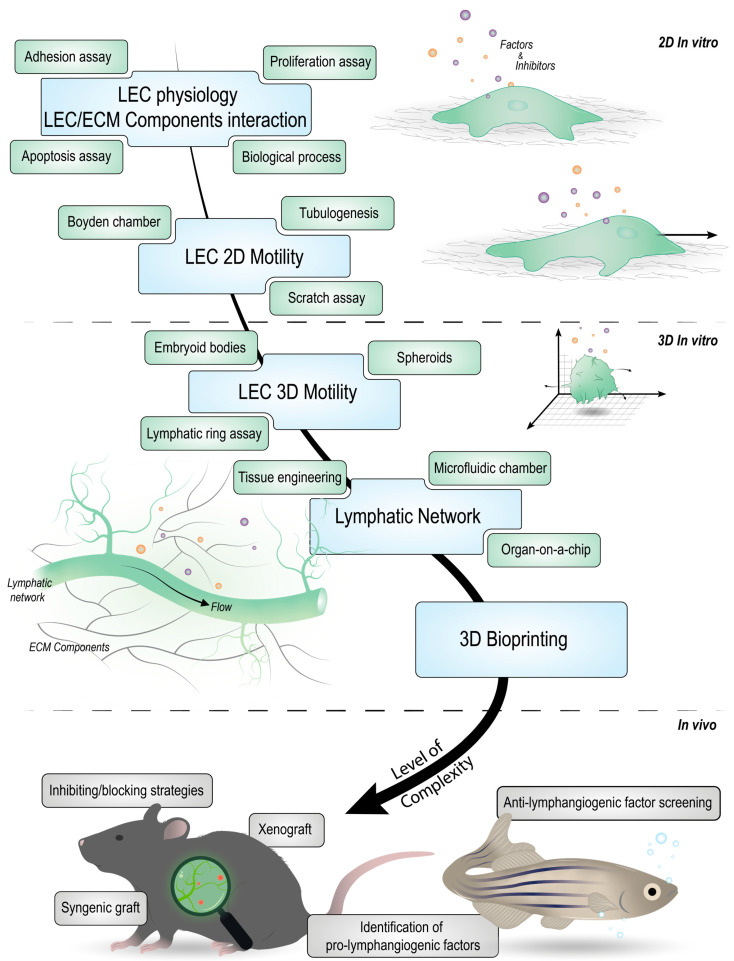
Summary of the in vitro and in vivo lymphangiogenesis (LA) assays according to their level of complexity. The first level of complexity refers to 2D in vitro cultures of isolated LECs and is used for investigating any individual step of the lymphangiogenic process (proliferation, migration, invasion, etc.) and morphogenesis. Three-dimensional in vitro static cultures increase the level of complexity and enable the study of the biological mechanisms underlying the whole process of LA. The third degree of complexity relates to in vitro 3D cultures, including flow and engineered constructs. In vivo mouse and zebrafish models stand for the highest level of complexity.

In vitro models are suitable for investigating the mechanisms underlying lymphangiogenesis under fixed experimental conditions. However, no in vitro models are currently capable of mimicking the entire process of LA, only parts of it. Figure 2 brings together the different models mentioned here, classifying them according to their level of complexity. Table 1 classifies the different cited models according to their pros and cons.

### 3.1. Two-Dimensional Cultures (2D Cultures)

LECs are grown in monolayers directly on culture plates or on matrix-coated plates [66]. These LEC monocultures are the basis of the in vitro tests to study LA. Each step of this process can be investigated through different assays: proliferation, migration, invasion, adhesion, or tubulogenesis tests. Apoptosis can also be studied, as well as cell cycle. The migration tests include the Boyden chamber and the scratch assay, in which the cells are in a monolayer and then scratched. The cells are cultured in a medium containing (lymph-)angiogenesis stimulators or inhibitors [67]. Combining bioinformatics, computer modeling, and imaging approaches, Williams et al. also studied 2D in vitro migration of endothelial cells. More particularly, they investigated which siRNAS of a big dataset control the migratory response of LECs and/or BECs [68].Tubulogenesis is applied to estimate the ability of cells to form vessels/tube-like structures on a collagen matrix (or Matrigel) in the presence or absence of experimentally defined molecules [69]. A limitation of this test is that the model does not represent what happens in vivo as LA takes place from already existing vessels. These 2D LEC monocultures allow the study of independent functions but do not consider more complex biological processes, such as, for instance, endothelial cell activation at the onset of LA, branching, and the ECM remodeling. Therefore, to overcome this lack, this research is more focused on the 3D study of LA. 

### 3.2. 3D Static Cultures

Growing lymphatic capillaries in vitro as 3D structures remains an important challenge to mimic the real in vivo situation and to better understand the complex LA process [70]. Moreover, the exploration of the lymphatic system has highlighted the importance of the physical (matrix stiffness and flow) and biochemical (matrix composition, soluble factors, and cellular components) in LA. Therefore, the emergence of models including this parameter, as well as 3D study, is essential for a further understanding of LA [71].

LECs derived from human embryonic stem cells allow an easier implementation of 3D cultures and can be easily grown as embryoid bodies embedded in a matrix [41]. LEC spheroids enable the investigation of migration, proliferation, or lumen formation in a more accurate 3D environment and the interaction with a thicker and stiffened matrix [72]. The spheroids are surrounded by a type I collagen matrix permitting the study of ECM remodeling. In addition, this technique enables the use of genetically modified LECs and LA stimulators or inhibitors [41]. However, the spheroid model is not adapted for the study of mature lymphatic vessels. In addition to spheroid analysis, the lymphatic ring assay technique has been validated as a model to provide information on the mechanisms of LA. It is based on the aortic ring test, a widely used model for the study of angiogenesis. Genes and molecules involved in LA can be identified through this 3D lymphatic culture. Lymphatic vessel fragments are harvested from the murine thoracic lymph duct and embedded in a collagen gel [70].

### 3.3. 3D Cultures Including Flow

To get closer to in vivo models, Ng et al. have highlighted the importance of the interstitial flow on LECs in in vitro models. They developed a 3D model to mimic and approximate in vivo models by inducing artificial flow in collagen gel containing LECs [73]. The interstitial flow differentially stimulates BEC and LEC morphogenesis in vitro. In order to couple the lymphatic regeneration process by combining interstitial fluid flow events and LEC migration, Boardman et al. created a skin regeneration model implanted in mouse tails and showed that lymphatic cell migration is initiated in the direction of the flow [74]. This model opened a new door to understanding the importance of interstitial flow on LECs but also highlighted a significant point: the matrix composing the model. Indeed, Helm et al. pointed to the composition of the matrix as an important parameter to consider when modeling tissue reconstruction and in vitro 3D modeling. They were able to form and regenerate lymphatic capillaries in collagen and fibrin matrices with a certain percentage and applied interstitial flow to them [75]. Subsequently, there has been an emergence of models on interstitial flow, including the model described by Pisano et al., who multiplied the forces of flow and thus developed microfluidic systems corresponding to the size of biological systems [76]. These tools are essential and allow a more in-depth study of the lymphatic microenvironment. They have thus combined a standard Boyden chamber with a microfluidic system to simulate the mechanical actions found in vivo. Beyond the purely mechanical study, this model also allowed the study of cell transmigration through a layer of LECs. This additional information enabled the study of the role of the flow on the penetration of cancer cells through an LEC monolayer. This study adds an additional dimension, coupling the study of LA and cancer [76].

In parallel, other ex vivo models have emerged in tissue engineering and allow the further integration of new parameters. One of the first engineered models established by Marino et al. enabled the formation of lymphatic capillaries in fibrin and collagen hydrogels. This study evaluated the optimal concentration of the matrix and, once the in vitro 3D construction was completed, the model was integrated in rats and in skin grafts [77]. In the interest of investigating the microenvironment of the lymphatic system and the different mechanisms of regulation of the lymphatic network, the microfluidic system, an organ-on-a-chip, has proven to be interesting in the modeling of the microenvironment from a dynamic point of view. These microfluidic systems have been used to characterize the lymphatic barrier in normal and pathological conditions [76]. Frenkel et al. developed a 3D microfluidic lymphatic vessel model, in which they study the interaction of LA with cancer organoids [78]. To add parameters to the interstitial flow, Kim et al. used the microfluidic platform system to reconstruct lymphatic budding [79,80]. Gibot et al. generated an in vitro tissue-engineered 3D human lymphatic microvascular network that includes the co-culture of LECs and fibroblasts, allowing the study of 3D lymphatic vessel branching, vascular permeability, and blind-ended junctions, but also creates a reproducible and interesting system for testing LA modulators [69,80,81]. In addition, Osaki et al. developed a device containing microchannels to evaluate the effects of different drugs on LA [80,82]. These microarray models remain innovative and are a real tool in understanding the mechanisms of LA. However, it is important to incorporate more biological components of the microenvironment to advance the understanding of tissue interactions in normal but also in pathological conditions. More information about the best practices for engineering new microvascular networks on-chip in the context of LA can be found in the paper of Tronolone and Jain [83].

To complement these models, 3D bioprinting has made possible the development of in vitro 3D constructs of LA in a tumor context in artificial matrices. In such systems, LECs are seeded in a sacrificial bioprinting matrix with tumor cells encapsulated in a hydrogel [80].

### 3.4. In Vitro Models—Discussion

All these studies allow a further understanding of the importance of 3D LA modeling, but these models should be definitively improved by considering all the other parameters involved, such as the ECM, the microenvironment, and their involved cellular and molecular actors, including collagen fibers and fibroblasts. Even with these features included, these in vitro models are still preliminary and need to become more complex to get closer to the in vivo observations. Currently, the transition between in vitro and in vivo can be seen in the study of Landau et al., in which engineered constructs of human lymphatics were developed. These constructs are incorporated in vivo in mice, resulting in anastomose with the host lymphatic vessels [84]. At present, in vivo studies/models remain essential for a relevant exploration of the physiological systems, including the lymphatic one.

In vitro modeling displays many advantages, including low cost, a completely controlled environment, and the absence of ethical issues. However, to study and understand cancer, which is a complex and multifactorial disease, it is essential to consider several parameters, such as the tumoral microenvironment and the inflammation. Such variables are reachable in in vivo models and make them powerful and complementary allies of in vitro in the study of tumoral processes such as LA.

## 4. In Vivo Models

In order to understand the complex and multifactorial process of lymphangiogenesis in cancer, in vivo models have added a dimension and a level of complexity to in vitro models (Figure 2). Several in vivo LA models are available and used to study the physiological state of this process but also to understand LA in a particular pathological context, such as in cancer [45].

### 4.1. Mouse

As a model for human cancer, the mouse has already proven to be an unavoidable research tool thanks to the genetic and physiological homology with human tumors [85]. As a recipient for tumor cells and/or genetic engineered organisms, the mouse largely contributes to a better understanding of tumorigenesis and its associated hallmarks, including LA. The transplantation of tissue or tumor cells derived from a donor of a different species from the recipient (xenograft) is a predominant approach in cancer modeling. In these model types, human tumor cell lines can be implanted in immunodeficient mice in order to induce tumorigenesis and further study the associated features. Several studies aim to study tumor LA by implanting human tumor cell lines and investigating their impact on the lymphatic network [86,87,88].

Developmental studies of the lymphatic vasculature have already contributed to the identification of key lymphangiogenic actors. Indeed, essential agents such as VEGF-C, VEGF-D, or VEGFR-3 were found to be crucial during normal mouse development [89]. The study of eventual key lymphangiogenic factors can be performed by using the matrigel plug assay, which consists in subcutaneously implanting gel containing compounds to test [90]. Xenograft-transplanted mouse models have demonstrated their usefulness in confirming the implication of these identified molecules in LA and cancer dissemination. The importance of VEGF-C-induced LA in cancer progression was largely studied in xenograft mouse models. The overexpression of VEGF-C by solid tumors was shown to increase peritumoral and intratumoral lymphatic vessels, as well as metastasis formation [91,92,93,94]. Moreover, when the RipVEGF-C transgenic mouse strain was crossed with the Rip1Tag2 strain, which is known to generate non-metastatic pancreatic beta cell tumors and VEGF-C-induced LA around the pancreatic beta cells, and promote metastasis in regional lymph nodes [92,95]. In other comparable studies, transplanted VEGF-D-overexpressing tumor cells were shown to promote tumor LA and increase the metastasis rate via dilatation of the collecting lymphatic vessels [96,97].

The ability of xenografts to reproduce the tumoral cascade and to generate a remodeled and extended lymphatic network also opens the way to evaluating anti-lymphangiogenic strategies through inhibitory or blocking compound screening. Such approaches can be used in order to elucidate or confirm the pro-lymphangiogenic role of a protein and even to participate in the emergence of new potential treatments. Several studies have already demonstrated an anti-lymphangiogenic effect by using blocking antibodies [96]. Because of their predominant role in lymphatic vessel scaffolding, VEGF-C and VEGF-D axis constitute ideal targets. Anti-VEGFR-3 antibodies, as well as soluble VEGFR-3, which competes with the endogenous receptor and traps VEGF-C/-D, showed a deleterious effect on tumor LA and metastasis in transplanted mice [86,98,99,100]. Antibodies targeting ephrinB2, a ligand of the EphB4 receptor, displayed a lymphatic vessel number decrease in transplanted mice [101], whereas antibodies against Neuropilin-2 reduced the tumoral LA, in addition to leading to a decrease of the metastasis number in the LN and distant organs [102,103]. The blocking of the ANG2/TIE2 pathway demonstrated an inhibition of lung and LN metastasis via an improved endothelial cell integrity [87]. Pharmacological compounds have also demonstrated an anti-lymphatic and anti-metastatic activity in a breast cancer mouse model [104]. More recently, the efficacy of afatinib, an EGFR tyrosine kinase inhibitor, was demonstrated in a lung adenocarcinoma HCC287 xenograft mouse model, where the tumor growth was inhibited and the lymphatic densities as well as the vessel diameter were decreased [105].

Besides xenografts, syngeneic transplants also present advantages for cancer cascade investigation. In this type of experiment, murine tumor cell lines are implanted to generate a solid tumor in order to further study the mechanisms of cancer. Several murine cell lines derived from spontaneous or chemically induced cancer are described for angiogenesis, metastasis, or LA modeling. For instance, Lewis lung carcinoma, CT26 colon carcinoma, 66cl4 mammary carcinoma, and B16 melanoma cells showed their ability to induce LA in several studies [106]. These models have the advantage of using immuno-competent and transgenic mice and allow the investigation of the remodeling occurring in draining LNs after tumor cell transplants. The ear sponge assay is an easy and reproductive model. In this system, a gelatin sponge soaked with tumor cells is implanted between the two mouse ear skin layers for 2–4 weeks in order to induce primary tumor growth. This model allows the tumor-associated LA study as well as the mimicking of the metastatic cascade in tumor draining LNs, thus making possible the characterization of the remodeled sentinel LN at the pre-metastatic and metastatic state [107,108].

### 4.2. Zebrafish

Due to their physiological and genetic similarities with humans, zebrafish constitute another powerful biological tool, which has already contributed to science advancement. Its high fecundity and the low cost of maintenance make it an ideal actor for disease modeling. Indeed, thanks to its ability to grow rapidly and its transparency during the early stages of life, it is ideal for development study [109], in addition to being largely used in genomics [110,111]. More recently, the use of zebrafish in cancer research became the aim of several studies and reviews [112,113]. Indeed, its properties allow scientists to easily monitor in vivo tumor growth, to perform large drug screening, and to investigate cancer-associated features such as angiogenesis and LA [114,115]. Thanks to the lack of a competent adaptive immune system during the early stages of life and to the apparition of immune-deficient zebrafish strains, xenograft is also an option for cancer study [116]. Recently, Chen et al. have reported an elegant update of zebrafish xenograft models in cancer research [112]. It is possible to transplant and to monitor tumor cells both in embryonic and adult animals. Indeed, the native embryonic transparency and the generation of transgenic transparent adult zebrafishes in 2008 open the way to an easier monitoring of in vivo tumor growth and cancer cell dissemination [117,118]. The resulting transparency makes it possible to clearly visualize the transplanted xenograft and to track labelled fluorescent tumor cells as well as extracellular vesicles in vivo [119,120].

Due to constant technical progress, zebrafish also became a powerful tool for in vivo imaging of blood and lymphatic development [118,121]. The generation of transgenic zebrafish lines that express fluorescent labeled vasculature enabled high-resolution real-time imaging of vessels [122]. In the zebrafish model, tumor neovascularization is so far the most studied. The implication of blood vessels in tumor spreading was already characterized in this model. Indeed, xenograft-induced neovascularization and the resulting dissemination of fluorescent labeled tumor cells were described [123,124]. The zebrafish is still underused as a model for tumor-induced LA [125]. To our knowledge, there is no zebrafish model yet that has proved its suitability for investigating LA in a tumoral context. Indeed, to date, zebrafish is essentially used for lymphatic vessel development study, the associated factor identification, and anti-lymphangiogenic molecule screening. In this model, the pro-lymphangiogenic activity of actors such as VEGF-C, VEGF-D, and YAP1 in lymphatic system growth and development was characterized [126,127,128,129,130]. The elaboration of therapeutic strategies, including the design of specific inhibitors, indeed represents a huge fraction of the zebrafish usage and essentially targets tumor angiogenesis. The development of anti-vascular drugs in zebrafish is mainly based on the combination between transgenic line availability and high-end imaging techniques [122]. Several tested compounds displayed an anti-lymphangiogenic effect. The formation of the thoracic duct in zebrafishes was prevented by the kaempferol, leflunomide, cinnarizine, and flunarizine [104]. As a result of this screening, the use of kaempferol displayed a reduction in tumor-associated lymphatic vessels and LN metastases in a breast cancer xenograft mouse model. In addition, a VEGF-C-VEGFR3-Erk pathway blocking strategy exhibits an anti-lymphangiogenic response [131].

For several years now, the zebrafish has demonstrated its usefulness in the discovery of new compounds by screening, strengthening the fact that it is a specialized system with unique research possibilities in in vivo cancer research alongside the mouse. Even if the study of tumor-induced LA in zebrafish is not as common as it is in mouse models, the interest in this topic and the availability of imaging techniques might lead to these models becoming important in gaining tumor LA understanding, as well as in future treatment development.

### 4.3. Imaging

Several animal models are now available to mimic the tumoral process and further study the multiple associated features. However, to measure and assess the impact of the tumor and tumor-derived factors on biological structures such as the lymphatic compartment or even the effect of drugs and inhibitors, researchers have to be able to visualize and quantify this network. That is where in vivo imaging techniques are needed. 

In fundamental research, it has become possible to image lymphatic vessels by exploiting the specificity of some LEC markers (described above, see Section 2) thanks to transgenic mouse strains and a better monitoring of proteins with fluorescent properties. Through the years and studies, several constructs implicating Prox1 were designed: Prox1-GFP, Prox1-tdTomato, Prox1-mOrange2, BACTg(Prox1-EGFP), and Prox1-EGFP BAC [132,133,134,135,136]. For LYVE1 and VEGFR-3, the following reporter protein constructs are described: Lyve1CreERT2_tdt_, Vegfr3_EGFPLUC_ and Vegfr3-YFP [137,138,139]. A more detailed list of these fluorescent lymphangiogenic reporters was addressed in 2018 in the review of Susan et al. [140]. Regarding transgenic zebrafish strains, there is no zebrafish model investigating tumor-associated LA, as said above. Transgenic fish lines nevertheless exist to study LA in other contexts [104,141,142,143]. Combined with tumor models, the latter could enable the zebrafish to become an interesting tool and a reference for LA in the context of tumor progression.

Regarding the (pre-)clinical (live) imaging of the lymphatic vasculature, non-invasive and sensitive visualization of the lymphatic system and vasculature is of paramount importance for diagnosis, treatments, and surgery. However, unfortunately, it remains challenging and less studied than the imaging of its blood counterpart [144,145,146]. This gap could be explained by the intrinsic properties of lymphatics, such as their size making the insertion of sensors difficult and the filtrating LNs only allowing local and not global contrast. Nevertheless, different methods and technologies enable imaging the lymphatic system and visualizing the process of LA, each with various invasiveness and resolution properties [147]. For example, with its high affinity for β-lipoproteins, Indocyanine Green possesses the property to accumulate in the lymphatic vessels, considering the high protein content of the lymph. This fluorescent dye is therefore commonly used for imaging the lymphatic system. Indeed, Near-Infrared Fluorescence imaging following Indocyanine Green injection is exploited in real-time during surgery [148] or for studying lymphatics-related processes, such as the lymphatic drainage in breast-cancer-related lymphedema [149], but also the LA impact during wound healing, injury repair [150], and arthritis [151]. Immuno-positron emission tomography with a radiolabeled lymphatic-specific antibody against LYVE-1 enables the imaging of tumor-induced LN LA [152]. The notion of the LN pre-metastatic niche is now well established and involves changes of the LN environment in order to be receptive to cancer metastatic cells [153]. The lymphatic remodeling occurring prior to the arrival of cancer cells, positron emission tomography combined with a lymphatic instead of a cancer marker, is more relevant as a prevention or diagnosis tool. Magnetic Resonance Imaging is another imaging technique used to investigate and visualize LA in various malignancies. For example, Yang et al. combined anti-podoplanin antibodies targeting lymphatic endothelium with GoldMag nanoparticles as a contrast agent and water-soluble polyethylene glycol to increase the stabilization and biocompatibility of the nanoparticles in order to evaluate breast cancer LA [154]. Other examples of in vivo imaging technologies of the lymphatic system are discussed by Polomska and Proulx [155] and by Elshikh et al. [156], the latter focusing on different oncologic imaging techniques of the lymphatic system.

### 4.4. In Vivo Models—Discussion

It is clear that animal models are an important tool in the modeling of cancer and the associated LA and have a place alongside in vitro experiments. Usually, in vitro and in vivo studies require imaging visualization and processing for quantification of the results. Additionally, in silico modeling, an emergent field of science, is about to become unavoidable for a further understanding of such complex biological mechanisms as LA.

It should be noticed that imaging is one of the prime ways to connect the in vivo and in silico parts. Through bioinformatics approaches, the multi-omics data from in vitro and in vivo models can be analysed, connecting again biological and engineering techniques. Indeed, the observations of the results of in vivo models can serve as input for the computational methods, which in turn are suitable for providing additional insights over and above what can be imaged.

**Table 1:** Advantages and disadvantages of the different cited lymphangiogenesis in vitro and in vivo models.

	Applications	Models	Advantages	Disadvantages	References
2D in vitro	LEC physiology LEC/ECM component interactions	Adhesion assay	- Low cost tests	- Inability to model the environment	[66,67]
		Proliferation assay			
		Biological process	- Rapid and easy observations		
		Apoptosis assay			
	LEC 2D motility	Boyden Chamber	- Easy to perform and to quantify	- Only 2D Migration	
		Scratch Assay			
		Tubulogenesis	- Self-organization - Observation of pseudo-vessel architecture	- No distinction between different phenotypes - Limited survival - No flow	[67,69]
3D in vitro	LEC 3D motility	Embryoid bodies	- 3D culture - Differentiation between tip and stalk cells - Possibility of lumen formation	- No flow - High volumes used for testing - No spatial control of gradients	[41,71]
		Spheroids			[41,71,72]
		Lymphatic ring assay			[71,72]
	Lymphatic network	Microfluidic chamber	- Integration of gradients and flow - Faster lumen formation similar to embryogenesis	- Problem of standardization	[71,73,74,75,76,77,78,79,80]
		Organ-on-a-chip			
In vivo	Animal models	Xenograft	- Use of human cells	- No impact of immunity in immunosuppressed animals	[71,89,90,91,92,94,95,96,97,98,99,100,101,102,103]
		Syngenic graft	- No rejection - Immunocompetent animals	- Use of cells with the same genetic background than the host - Inability to use human cells	[104,105,106]
		Zebrafish	- Pro- and anti-lymphangiogenic factor screening - Developmental studies	- Difficult for studying cancer-associated lymphangiogenesis	[102,124,128,129]

## 5. In Silico Models

Defined in analogy to in vitro and in vivo, the term in silico refers to the work performed using mathematical modeling and computer simulations. In silico approaches are increasingly applied in fundamental cancer biology research, focusing on, amongst others, tumor angiogenesis and lymphangiogenesis [46]. Not all biological features can be studied in in vitro and in vivo models as only a few components can be investigated at the same time, representing one of the limitations of these models. In silico models have a remarkable integration capacity, enabling not only a better understanding of the different actors individually involved in LA but also their interactions. They offer new perspectives and allow the generation of new hypotheses and predictions that are not always straightforward to verify experimentally due to practical, financial, ethical, and timing constraints. Moreover, they can guide scientists towards more informative experiments. Indeed, the in silico techniques coincide with the principle of the 3 Rs: reduction by better planning the experiments, refinement by generating modeling methods to finer extrapolate experimental data, and replacement by enabling a more accurate translation from animal to human experiments [48]. In silico models are already used during research and development phases, as well as for clinical trial design and optimization [49]. Regulatory guidelines and standards are becoming available, providing guidance for the verification and validation of in silico methods for clinical and industrial uptake [157,158].

The next paragraphs explain the in silico pipeline and discuss the different existing mathematical and computationnal models related to lymphatics. Although many precise types of modeling techniques are specified hereafter, their subsequent mathematical and computational details will not be addressed, as this is beyond the scope of this review. The diverse ways to classify in silico models and tools in a biological context are reviewed in Appendix A. More information about the specific mathematical formalism (in a biological context) and the in silico pipeline can be found in Bekisz and Geris [159] and in Lesage et al. [160].

### 5.1. The In Silico Modeling Pipeline

The modeling procedure and the development of models, which can be sorted according to different features and mathematical formalism (see Appendix A), follow a very precise pipeline divided into several distinct steps [161,162]. Specifically, the subject to be addressed and the questions to be asked of the model are first identified and clearly defined. 

A literature review is then conducted to report all the current data and knowledge about the topic, resulting in the choice of the best modeling formalism for the objectives to be achieved. The model formalism also depends on the biological data availability or at least the possibility to generate these data. A trade-off must be found between an accurate representation of the system under study and the multiple assumptions imposed because of the biological uncertainties and the limited experimental data provision. In this respect, hypotheses are not to be confused with details, whose level has no relation with the model accuracy but rather with the model capacity to answer the stated problem. Let us quote the relevant sentence of Manfred Eigen: “A theory has only the alternative of being right or wrong. A model has a third possibility: it may be right, but irrelevant”.

The next modeling steps are the mathematical formulation, followed by the computational implementation, because only a few models can be solved purely analytically. The models are often composed with variables and parameters, most of which are not known and must be estimated. Parameter estimation is therefore requested to calibrate the model and consists in determining the optimal parameter values to enter into the model so that the generated output fits as best as possible with the biological data, generated previously or for the purpose.

Once the model has been calibrated, it is simulated with efficient algorithms and the in silico outputs are provided. Wrong results are not necessarily related to a wrong mathematical formalism but can also be linked to false starting hypotheses or issues with the data used for calibration (garbage in, garbage out).

Coherent model predictions can be validated with new experiments or with previously generated biological observations. Calibration and validation usually do not use the same datasets. Depending on the divergence between the predicted and the observed data, the model might be subjected to modifications and undergo several improvement and optimization cycles for refinement. In addition to reproducing data from biology, the last optimized version of the mathematical model is therefore considered as a tool that can be used to test new experimental conditions.

**Figure 3:**
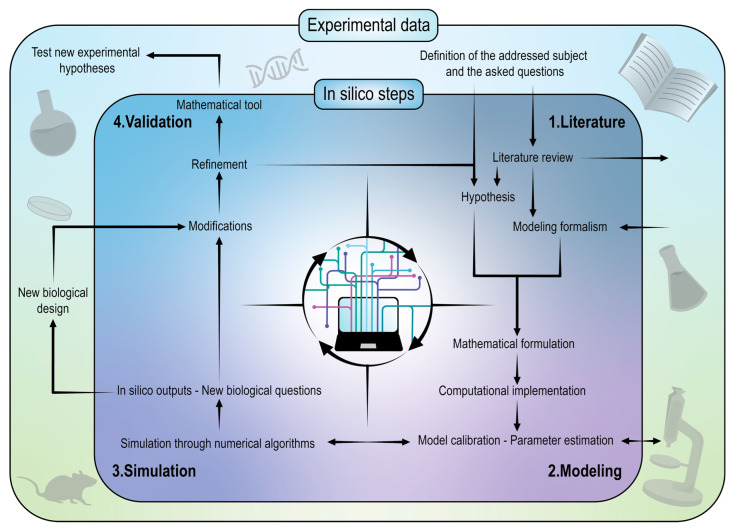
The mathematical modeling pipeline in biology. The in silico procedure is divided into 4 distinct steps, including literature, modeling, simulation, and validation. During this entire modeling process, the symbiotic approach comparing in vitro and in vivo experimental data with computer outputs is used.

It is important to realize that in silico models (like all other model systems) are not self-sufficient but are constructed, validated, and iteratively refined through in vitro and in vivo experiments before being sufficiently precise and ready to provide insightful outputs. The credibility of in silico models can be established for a precisely defined context of use, by applying the so-called VVUQ (Verification, Validation, and Uncertainty Quantification) [163]. Figure 3 gathers all the steps outlined above regarding the modeling pipeline and highlights the symbiotic approach, mixing the in vitro, in vivo, and in silico methods.

### 5.2. In Silico Models of LA

For several years, the impact of the vascularization on tumor progression has been intensively studied through mathematical modeling, especially the influence of angiogenesis. Good reviews of in silico models for angiogenesis and its role in tumor progression were written by Peirce [164], Heck et al. [165], Chaplain [166], Scianna et al. [47], Mantzaris et al. [167], Levine et al. [168,169], Qutub et al. [170], Suzuki et al. [171], and Lowengrub et al. [172]. Even if some previously reported models of angiogenesis could be easily adapted for LA, this process presents particular features requiring specific dedicated models. Though considerably less widespread than for the process of angiogenesis, several mathematical and computational models have been implemented in the context of the lymphatic system and LA. Currently, very few in silico models specifically focused on tumor LA have been developed. However, the models and tools elaborated in ‘simpler’ lymphatic contexts will nevertheless be discussed in this review article, sorted by application domain, and can serve as a starting point for more focused tumor LA models.

#### 5.2.1. Lymphatic Flow

The principal initiator of the in silico lymphatic system modeling is Reddy, who computationally investigated, among others, the flow through terminal lymphatics but also the lymph circulation biomechanics and the valve physics [173,174,175,176]. Elhay and Casley-Smith were also pioneers and used normal laws of physics related to flow and diffusion to study the initial lymphatics [177]. MacDonald adapted one of Reddy’s models to study the lymph flow in the collecting lymphatic vessels [178]. Different modeling techniques of drainage in primary and collecting lymphatics, as well as lymphatic valves and nodes, are reviewed in the paper of Roose and Tabor [179]. Mozokhina and Savinkov highlighted the different mathematical models implemented to represent and study the lymph flow in the lymphatic system and its subunits (lymphangions, valves, and LNs) [180]. In this context, the majority of the developed models are built with zero-, quasi-one-, or one-dimensional approaches. Zero-dimensional methods, so-called lumped models, refer to the electrical circuit theory, comparing the pressure, lymph flux, and mass with voltage, current, and charge, respectively. Lymph flow is usually described in one-dimensional approaches with Navier–Stokes equations or with the law of mass and momentum conservation. Comparison between 0D and 1D formulation in the context of modeling lymph flow in the lymphatic system has been explained in the article of Tretyakova [181]. Cooper et al. used a finite element image-based model to investigate the fluid flow through the LNs [182]. The effect of a permeable interstitium on a network of initial lymphatics and pre-collectors was investigated by Ikhimwin et al. with a lumped-parameter model composed of differential–algebraic equations [183].

#### 5.2.2. Tumor Lymphangiogenesis

The pioneer in the field of mathematical tumor LA is Lolas, who developed in collaboration with Friedman a mathematical model of tumor LA through a system of partial differential parabolic equations [184]. By following the mathematical time and space evolutions of different key variables, such as the LEC and tumor cell densities, the effect of potential anti-cancer drugs has been studied through this model, also considering the effect of the ECM and its components [185]. This model has been improved by adding the proteolysis effect, enabling the highlighting of the influence of the proteolytically and un-proteolytically processed growth factors on tumor dissemination and LA [186]. 

#### 5.2.3. Cellular Interactions in Lymph Nodes

Novkonic et al. investigated LNs and reviewed the different computational models of LNs, especially investigating the interactions between stromal and immune cells [187]. Indeed, because of their strategic position, their filtration capacity and their link with the immune system, LNs are also widely represented mathematically. Some key regulators of the chemokine gradient formation in LNs could have been predicted by the in silico model developed by Jafarnejad et al. [188]. Differential equations representing biochemical reactions were combined with well-known fluid mechanics rules describing lymph flow. Benchaid et al. developed two mathematical models for studying the interactions between cancer and immune cells in the LN [189]. The first model, focusing more on the interactions between cancer and immune cells, represents the spatiotemporal evolutions of proliferating tumor cells, dormant tumor cells, immune cells, and growth factors. The second model, complementary to the first, employs a hybrid multiscale approach, combining continuous and discrete modeling, representing the secondary tumor growth in the LN particularly. In addition to returning the three well-known regimes of tumor growth in the LN (tumor elimination, cancer-immune equilibrium, and tumor evasion) with specific parameters, the numerical simulations suggested that anti-PD-1/PD-L1 therapies could be more effective in the presence of high EGF concentrations in LNs.

#### 5.2.4. Blood and Lymphatic Vessel Interactions

Wu et al. integrated blood and lymphatic vascular systems in a hybrid continuous-discrete mathematical model of tumor growth to better elucidate the influence of interstitial fluid pressure and flow [190]. Their in silico model fitted with already proven biological experiments and generated new suggestions on the influence of the lymphatics in a tumoral environment. Fluid exchanges between lymph and blood vessels in LNs were also investigated by Jafarnejad et al. with a finite volume-based model, confirming that changes in the inflow/outflow conditions seriously impact the LN microenvironment components and therefore modulate the immune response [191].

#### 5.2.5. Lymphatic Biomechanics

Galie and Spiker used a finite element method to computationally model the transendothelial lymph transport in primary lymphatics, including relevant biomechanics [192]. Other in silico models of the lymphatic system focusing on its biomechanical behavior are reviewed in the article of Margaris and Black [193] or in Nipper and Dixon [194].

#### 5.2.6. Lymphatic Electrophysiology

Ion fluxes in LECs were modeled by Behringer et al. on the basis of the well-known equations of Hodgkin–Huxley [195]. This model enables a better understanding of the underlying ionic mechanisms of lymphatic endothelial function compared to blood vessels. Remaining in the field of electrophysiology, Contarino and Toro studied the lymphatic dynamical contractions with one-dimensional modeling for collecting lymphatics combined with an electro-fluid-mechanical contraction model for dynamical contractions, which is based on the FitzHugh–Nagumo theory [196].

#### 5.2.7. Bioinformatics

In the context of increasing computational capabilities going together with the big data era, bioinformatics tools are essential for the treatment and analysis of these data. Transcriptomic and single-cell data from (lymph node) lymphatic vasculature [197,198,199,200,201], as well as metabolomic [202] and proteomic [203,204] data, have already been produced from experimental lymphatic set-ups. In combination with imaging techniques, Williams et al. also used bioinformatics approaches to study the impact of particular siRNAs on the 2D in vitro migration of endothelial cells [68]. Regulatory gene and protein networks can be inferred from these large-scale data libraries to study intracellular dynamics governing cellular behavior and identify druggable targets.

#### 5.2.8. Others

Even if out of the scope, we mention here the studies by Wertheim and Roose, who proposed a mathematical model of LA in zebrafish embryos [205], and by Bianchi et al. who investigated LA in the context of wound healing with ordinary differential equations, highlighting the importance of the relative proportion between TGF-β and VEGF-C, rather than their absolute values [206]. By means of their in silico models, the different suggested biological hypotheses behind the latter process could be sorted. Furthermore, Tretyakova et al. used computational geometry and network graph models to investigate the structure and topology of the lymphatic system [207]. In a purely schematic and visualization perspective, an interesting library of interactive 3D models representing the lymphatic system and its associated diseases was designed by the Leukemia and Lymphoma Society [208].

**Figure 4:**
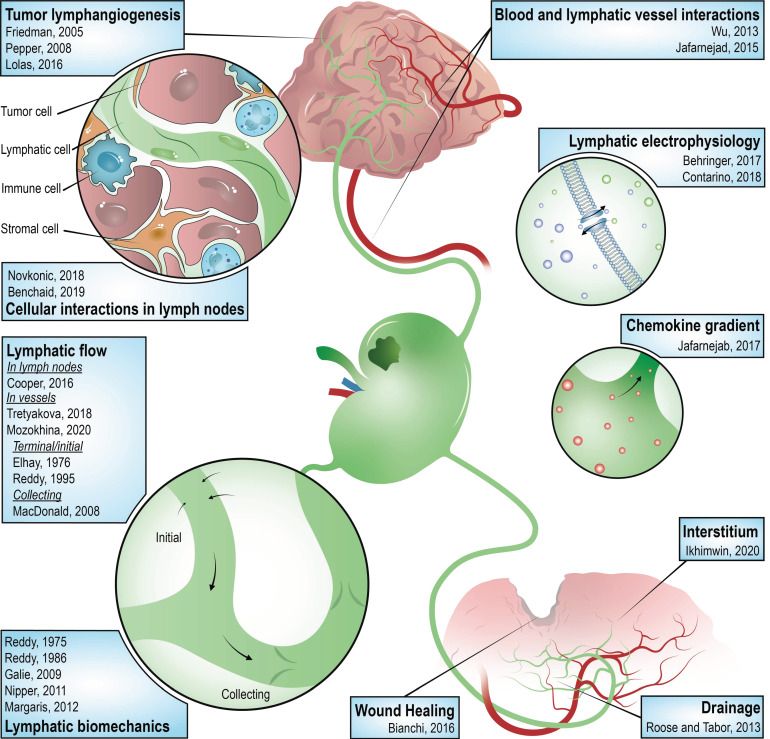
Summary of the in silico models developed in the context of the lymphatic system and the process of lymphangiogenesis. In the context of lymphatics, in silico models of lymphatic flow, drainage, and biomechanics were first developed. Tumor lymphangiogenesis was then studied with hybrid multiscale computational approaches, as well as the interactions between different populations of cells (tumoral, lymphatic, immune, and stromal cells). The effects of a permeable interstitium and a chemokine gradient on the lymphatic network were investigated through in silico techniques. Blood and lymphatic vessel interactions were studied with hybrid mathematical models. The well-known equations of Hodgkin–Huxley were used to mathematically investigate lymphatic electrophysiology. Skin wound healing was lastly modeled and studied with differential equations [173,174,176,177,178,179,180,181,182,183,184,185,186,187,188,189,193,194,195,196,206].

Figure 4 gathers together and illustrates the previously cited in silico models developed in the context of the lymphatic system and the LA process.

### 5.3. In Silico Models—Discussion

It is clear from the variety of in silico models cited above that there is a wide application area that can be covered by said models, in all phases of the R&D pipeline. In addition to the mostly knowledge-driven models cited above, bioinformatics tools are exploited for treatment and analysis of increasingly prevalent high throughput data. Finally, in complement to these in silico models acting as a digital twin of the lymphatic system or one of its features, computational analysis of biological images (often using machine learning or artificial intelligence) is another domain in which computer and mathematical methods are helpful in the context of lymphatic biology and clinical practice. The latter are also indispensable to the interpretation of the results of the models discussed in Section 4.

## 6. Perspectives, Limitations, and Conclusions

In a society where cancer is the second leading cause of death and where everyone has at least one relative affected by this disease, the improved understanding of its various aspects is of utmost importance [1]. Cancer cells notably disseminate and form metastases through tumor-induced vasculature, both blood and lymphatic vessels [3,4,5,6,7,8]. The process of lymphangiogenesis has nevertheless often been neglected in favor of the much-studied angiogenesis. However, its relevance in cancer and other pathologies is now well established. The LA study and the discovery of pro-lymphangiogenic drugs have been delayed as compared to angiogenesis, mainly due to the lack of adapted tools, and are now gradually increasing. This review provides a non-exhaustive up-to-date overview of the in vitro, in vivo, and in silico models of LA. These three types of modeling, each presenting advantages and limitations, are required to work all together in an effort to respect the 3R principle, including the reduction in animal experiments.

Low cost, tight control of the environmental conditions, and no ethical issues often make in vitro models very attractive, especially now that 3D cultures with flow and organotypic ex vivo approaches have been developed [69,76,77,78,79,81,82,83]. Unfortunately, most in vitro models insufficiently integrate different cell types in a relevant ECM, although that is known to play an essential role in both cancer and LA. In this context, cell-cell and cell-matrix communications are becoming of particular interest as they are at the heart of cell adhesion, migration, and differentiation. Fibroblasts would exert traction forces on the ECM, inducing signals potentially detected by cells sharing the same environment [209]. Changes in the alignment of collagen fibers in the ECM have been demonstrated in various pathologies, including cancer. The traction of collagen fibers by fibroblasts would impact the migration and invasion of cancer cells. The LEC–fibroblast interactions are therefore of paramount importance and worth considering when studying tumor-induced LA. Still in this context, it could be useful to investigate in depth the heterogeneity/plasticity of fibroblasts and LECs and also how they interact with the surrounding interstitial ECM.

In vivo approaches enable the better modeling of complex and multifactorial diseases such as cancer with a systemic integration of the tumoral environment and the inflammation. In the context of tumoral LA, transgenic mouse strains remain the favored animal model, both for their genetic and physiological homologies with humans and also for their ability to be monitored through proteins with fluorescent properties. Anti-lymphangiogenic drugs have even been investigated by means of xenografts reproducing the tumoral cascade with a remodeled and extended lymphatic network [98,99,100,101,102,103,104,105]. Because xenografts unfortunately often require immunodeficient mice to avoid graft rejection, approaches with syngeneic transplants have been developed, as in the ear sponge assay investigating the metastatic cascade in LNs [107,108]. Given a facilitated reproduction and decreased ethical constraints, zebrafish could putatively become a model of choice for exploring tumor-associated LA. However, the use of live animals for experimental purposes is increasingly questioned at an ethical level, and the development and uptake of alternative methods, including in silico approaches, are required.

The computational and mathematical contributions to biological fundamental research are no longer to be proven. Although not numerous, in silico models of tumor LA, including the ECM, have been developed and enabled to examine the effect of potential anti-cancer drugs [187]. These models should now be combined with the ones on LNs to have a more integrated and systemic approach to the metastatic cascade. As digital evidence, i.e., the results of computer-based methods, is increasingly included in regulatory filings, a regulation framework is (being) established. A standard is already detailing how to build credibility for computer models used in the context of medical devices (V&V40) [158]. Regulatory guidelines regarding the introduction of in silico approaches in the pipeline for drug development (beyond the classical pharmacometric models) are also being established [210]. Additionally, in analogy to good clinical and manufacturing practices, a good simulation practice is being developed by the community as a way to guide proper model development from bench to bedside [211]. All these activities will allow the building of necessary trust in in silico approaches and allow them to take their place as a third source of data generation in biomedical sciences, next to in vitro and in vivo.

The symbiotic approach, mixing in vitro, in vivo, and in silico methods, is of great interest for modeling tumor-associated LA. Each kind of model brings different but complementary information. Whether it is because they are not often conclusive in clinical trials or because of ethical questioning, the animal experiments will be reduced and possibly even eliminated in the future. Alternative and advanced techniques require the combination of in vitro models, prospectively a suite of organ-on-a-chips, with in silico models, prospectively digital twins. In reference to the article of Ingber: “is it time for reviewer 3 to request human organ-on-a-chip experiments instead of animal validation studies?”, we aimed to demonstrate in this review that this also holds true also for in silico experiments or a combination of both [212].

## Data Availability

Not applicable.

## Appendix A. In Silico Models—Background

Complementary information about the diverse ways to classify in silico models and tools in a biological context is provided in this Appendix A. In silico models can be sorted according to different features, from the subject addressed, to the time and spatial scales modeled, and to the mathematical formalism used to model the problem.

### Appendix A.1. Addressed Subject

Mathematical modeling extends to many areas of daily life, such as chemistry, engineering, social science, physics, and economics, as well as biology. In biology, in silico modeling is exploited in (amongst others) immunology, drug toxicity, genomics, epidemiology, metabolomics, neurology, and, of course, cancer [159]. In each of these fields, different levels of details can be addressed. This list is far from exhaustive.

### Appendix A.2. Length and Time Scales

In biology, and in cancer particularly, different length and time scales can be considered and modeled, from the gene and protein levels to the population level [157]. Regarding the gene and protein levels, high throughput technologies helped to provide access to large amounts of genomic, transcriptomic, proteomic, and metabolomic data. Increasing the computational capabilities going together with the big data era, new bioinformatics analysis tools were developed to infer differential expression patterns and regulatory networks to highlight key actors of this disease frequently connected to genome changes. Single-cell mechanistic behavior, cellular interactions, and the influence of different external clues on individual cells can also be investigated through models, often with a mathematical approach called agent-based modeling. The tissue level focuses more on the influence of growth factors, the ECM, and the vasculature on a group of cells (cancerous here). This tissue context involves many multiscale actors with their own identity and size, making it common to use hybrid modeling, which mixes a discrete formalism to represent the cells of interest with a continuous one to depict the environment and the other molecular species and cells. The influence of endogenous and applied external mechanobiological forces in cells or a set of cells can also be explored in these two previously mentioned levels. The organ level refers to the studies highlighting the structure–function aspect and exploring, for example, the impact of a tumor on a specific organ or biological system (and vice versa). Patient and population levels grow in complexity and are two of the highest (relevant) spatiotemporal scales that can be considered. By integrating several of the aforementioned levels (depending on the question at hand), virtual patients with specific parameters and features can be generated and participate in the reduction challenge of the 3R principle in clinical trials [213].

### Appendix A.3. Mathematical Classification

Different mathematical formalisms can be used for modeling a system of interest, focusing on the inputs used as well as the representation of the system. First, models can be distinguished according to the way they represent the system, either in a continuous or discrete manner. Second, besides the assumptions and simplifications, in silico models can be fed with two distinct sorts of information: qualitative knowledge and quantitative data. When only physical principles and physiological knowledge about the studied system are used to implement the model, it is referred to as mechanistic, white-box, physics-based, or knowledge-driven modeling. Contrastingly, models built solely on experimental data are black-box or data-driven empirical models, in which links between the outputs and inputs are established without using prior knowledge. Good illustrations of data-driven model technologies are machine learning, deep learning, and artificial intelligence. However, in practice, many white box models include several empirical elements and black box models are increasingly considering existing knowledge, giving rise to the category of grey-box models which are derived from both previous knowledge and experimental records in various proportions.

Models can also be distinguished according to the way they represent the system, either in a continuous or a discrete manner. In continuous modeling, for which partial or ordinary differential equations are often used, the values of the involved variables belong to a continuum, whereas the variable values in discrete models are imposed from a discrete set and implemented through agent-based modeling. Hybrid approaches combine discrete modeling to represent cells of interest and continuous modeling for describing environmental factors and clues.
